# The complete plastome sequences of *Pseudowintera colorata* and *Tasmannia lanceolata* (*Winteraceae* – *Canellales*)

**DOI:** 10.1080/23802359.2020.1847620

**Published:** 2021-01-20

**Authors:** Sangjin Jo, Ki-Joong Kim

**Affiliations:** Division of Life Sciences, Korea University, Seoul, South Korea

**Keywords:** Plastomes, Canellales, Winteraceae, *Pseudowintera colorata*, *Tasmannia lanceolata*

## Abstract

In this study, we report the complete plastome sequences of two Winteraceae taxa, *Pseudowintera colorata* (MT555077) and *Tasmannia lanceolata* (MT555078). Both plastomes show typical quadripartite structure. The plastome size of *P. colorata* is 161,675 bp, which consists of 89,583 bp large single-copy (LSC), 18,606 bp small single-copy (SSC), and 26,743 bp inverted repeat (IR) regions. The plastome size of *T. lanceolata* is 160,424 bp, which consist of 88,589 bp LSC, 18,351 bp SSC, and 26,742 bp IR regions. Both plastomes contain 113 genes, including 79 protein-coding, 30 *tRNA*, and four *rRNA* genes. Sixteen genes contain one intron and two genes (*clp*P and *ycf*3) have two introns. Ninety-three and 89 simple sequence repeat (SSR) loci are scattered in the *P. colorata* and *T. lanceolata* plastomes, respectively. Our phylogenetic tree shows the relationship of (*T. lanceolate* (*P. colorata,* *Drimys granadensis*)) in the Winteraceae. The Canellales (incl. Winteraceae) are the sister group of Piperales.

*Pseudowintera colorata* and *Tasmannia lanceolata* are native to New Zealand and Southeastern Australia, respectively. They belong to the family Winteraceae in the order Canellales (APG IV 2016). Winteraceae consists of two subfamilies, five genera, and approximately 105 species (Christenhusz and Byng 2016). *Pseudowintera* and *Tasmannia* belong to the subfamily Winteroideae and consist of approximately three and 50 species, respectively.

The leaves of *P. colorata* and *T. lanceolata* were ground into powder in liquid nitrogen and total DNAs were extracted using the G-spin^TM^ IIp for Plant Genomic DNA Extraction Kit (iNtRON Biotechnology, Gyeonggi-do, Korea). Two voucher specimens were deposited in the Korea University Herbarium (KUS acc. nos. TN2020-0006 and TA2019-0017) and genomic DNAs are deposited in the Plant DNA Bank in Korea (PDBK acc. nos. 2020-0006 and 2019-0017). Two complete plastome sequences were generated using an Illumina MiSeq platform (Illumina Inc., San Diego, CA). De novo assemblies and annotations of plastomes were performed using the Geneious version 11.1.5 (Biomatters Ltd., Auckland, New Zealand; Kearse et al. 2012), National Center for Biotechnology Information (NCBI) BLAST, and tRNAscan-SE programs (Lowe and Eddy 1997). The average plastome coverage of *P. colorata* and *T. lanceolata* is 661× and 2006×, respectively. The simple sequence repeats (SSRs) were detected by the Phobos version 3.3.12 program (Leese et al. 2008) in the Geneious version 11.1.5. For the phylogenetic analysis, we selected and downloaded 20 related complete plastome sequences based on the APG IV system (APG IV 2016) from the NCBI database.

The plastome size of *P. colorata* is 161,675 bp, which consist of 89,583 bp large single-copy (LSC) region, 18,606 bp small single-copy region (SSC), and 26,743 bp inverted repeat (IR) region. The plastome size of *T. lanceolata* is 160,424 bp, which consists of 88,589 bp LSC region, 18,351 bp SSC region, and 26,742 bp IR region. They are similar to the *Drimys granadensis* (160,604 bp, NC008456) plastome reported in previous studies (Cai et al. 2006). They show a typical quadripartite structure. Both plastomes hold 113 unique genes, including 79 protein-coding genes, 30 *tRNA* genes, and four *rRNA* genes. Six protein-coding, seven tRNA, and four rRNA genes are duplicated in the IR regions. The average A-T contents of the two plastomes are 61.1% in *P. colorata* and 61.2% in *T. lanceolata*, respectively. Sixteen genes contain one intron and two genes, *ycf*3 and *clp*P, have two introns. A total of 93 SSR loci are distributed throughout the *P. colorata* plastome. Among these, 78, 10, and 5 are mono-SSR, di-SSR, and tri-SSR loci, respectively. A total of 89 SSR loci including 71 mono-, 14 di-, and 4 tri-SSRs are scattered in the *T. lanceolata* plastome.

To estimate the phylogenetic relationships of two species, we constructed a maximum likelihood (ML) tree using 22 basal angiosperm taxa. Phylogenetic analysis was performed on a data set that included 79 protein-coding genes and four *rRNA* genes from the 22 selected taxa using RAxML version 8.2.12 in CIPRES webserver (Stamatakis 2014) under GTR + G + I model with 1000 bootstrap replicates. The 83 gene sequences (79,005 bp in length) were aligned with the MUSCLE program using Geneious version 11.1.5 (Biomatters Ltd. Auckland, New Zealand; Kearse et al. 2012). The resulting tree supports the monophyly of three members of Winteraceae by 100% bootstrap value. The tree shows the (*T. lanceolate* (*P. colorata, D.granadensis*)) relationship within the Winteraceae. The Canellales (incl. Winteraceae) were the sister group of Piperales (Figure 1). This relationship is also supported by the previous studies (Karol et al. 2000; Doust and Drinnan 2004). The complete plastome is rare in the basal lineages of angiosperm. Only one sequence in Canellales was available before our report. Therefore, the complete plastome sequences of *P. colorata* and *T. lanceolata* will provide a useful resource for the evolutionary studies of Canellales.

**Figure 1.**
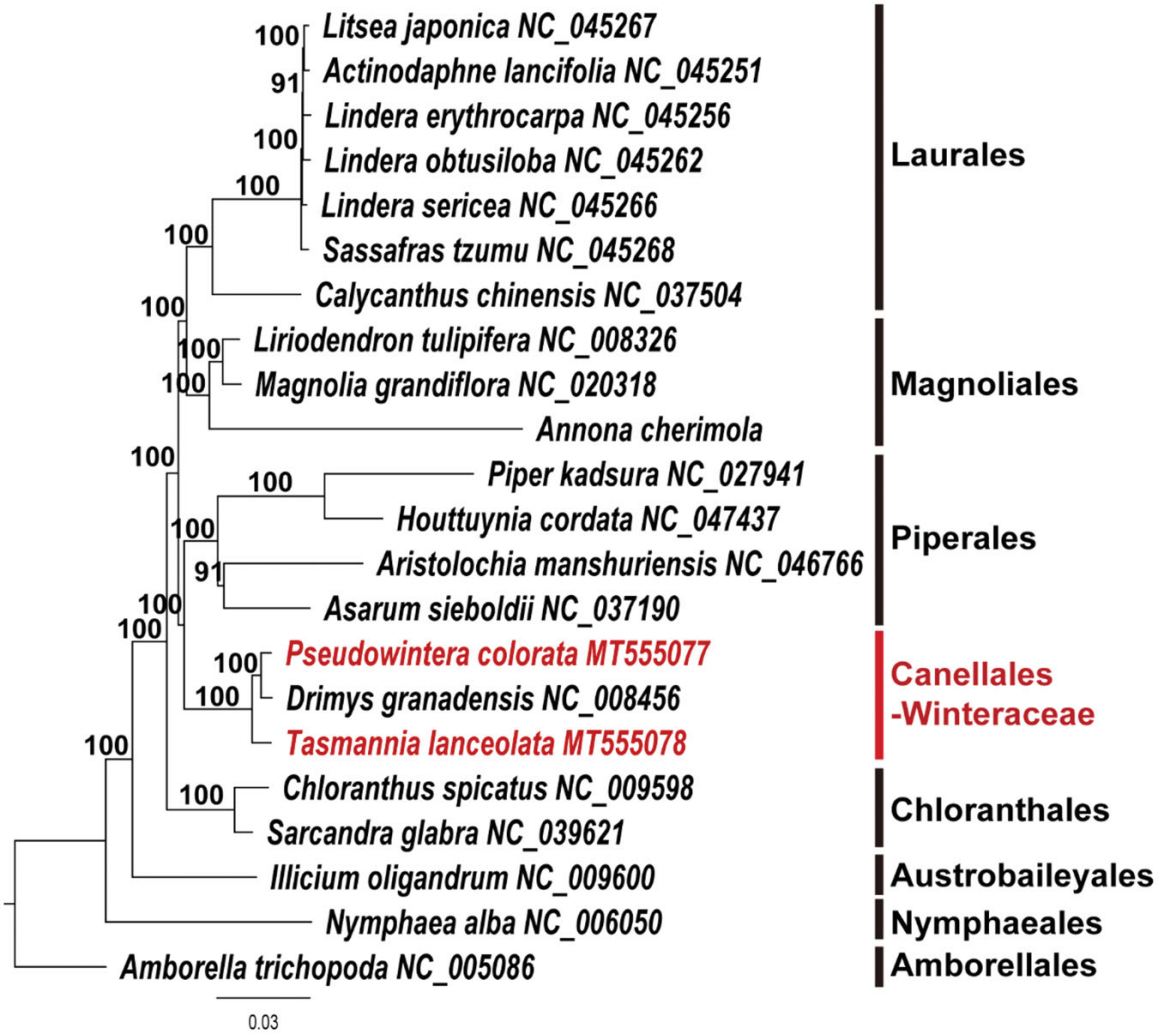
Maximum likelihood (ML) tree based on 79 protein-coding and four *rRNA* gene sequences from 22 plastomes of basal angiosperms as determined by RAxML(−ln *L=* 315655.165486). The numbers at each node indicate the ML bootstrap values.

## Data Availability

The data that support the finding of this study are openly available in GenBank of NCBI at https://www.ncbi.nlm.nih.gov, reference number MT555077 for *Pseudowintera colorata* and MT555078 for *Tasmannia lanceolata*.
